# The aging choroid plexus and its relationship with gut dysbiosis and Klotho decline: possible intervention strategies

**DOI:** 10.1007/s11357-025-01797-1

**Published:** 2025-07-22

**Authors:** Giovanni Lai, Lisa Bevilacqua, Maria Elisa Giuliani, Giorgia Bigossi, Serena Marcozzi, Tiziana Casoli, Pasqua Abbrescia, Antonio Frigeri, Marco Malavolta, Marta Balietti

**Affiliations:** 1 Advanced Technology Center for Aging Research and, Geriatric Mouse Clinic, IRCCS INRCA, Via Birarelli 8, 60121 Ancona, Italy; 2 Center for Neurobiology of Aging, IRCCS INRCA, 60121 Ancona, Italy; 3 Department of Translational Biomedicine and Neuroscience School of Medicine, University of Bari, 70100 Bari, Italy; 4 Department of Clinical and Molecular Sciences, Università Politecnica Delle Marche, 60126 Ancona, Italy

**Keywords:** Choroid plexus, Gut dysbiosis, Klotho, Neurodegeneration, Probiotics, Gene-based delivery strategies

## Abstract

The choroid plexus (ChP) is a complex ventricular structure that forms a semi-permeable barrier between the blood and cerebrospinal fluid (CSF). It is responsible for CSF secretion and clearance, contains macrophages, and is one of the few sites within the central nervous system (CNS) where T cells are present. Additionally, the ChP plays a role in detecting peripheral inflammation, which leads to the modulation of its epithelial cell function. Despite its critical importance in maintaining brain homeostasis, the ChP is often overlooked, particularly concerning the changes it undergoes with aging, such as reduced barrier function, impaired CSF production, and altered immunosurveillance. These age-related alterations may contribute to several harmful effects, including neuroinflammation and oxidative damage, potentially predisposing individuals to neurodegenerative conditions. Although knowledge is still limited, gut dysbiosis and decreased Klotho levels—of which the ChP is one of the main sources—appear to be significant contributors to ChP impairments. This narrative review will examine the impact of age-related gut dysbiosis on the CNS, focusing on the ChP, and explore the effects of reduced Klotho levels in this brain structure. We will also propose the hypothesis that combining the administration of probiotics capable of restoring gut microbiota eubiosis with gene therapy to upregulate Klotho in the ChP could help preserve the structural and functional integrity of the aging brain. Finally, we will provide a technical overview to ensure that vectors encoding Klotho cDNA achieve maximum specificity for the ChP, thereby avoiding off-target effects.

**Graphical abstract:**

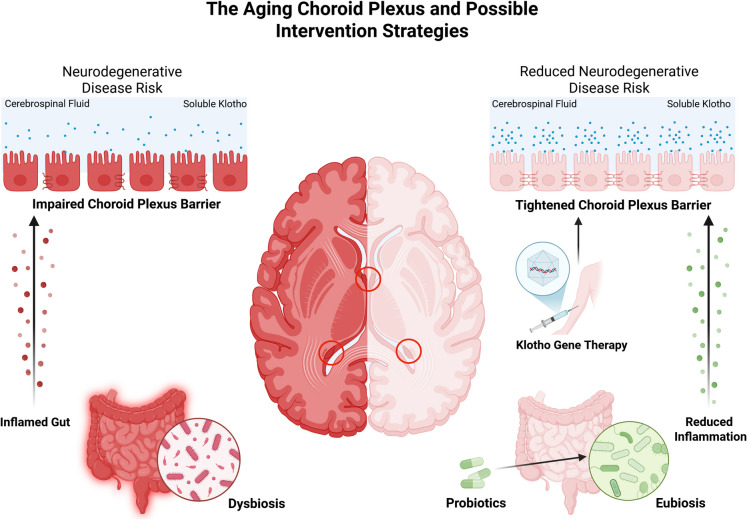

## Introduction

The aging global population is leading to a rise in age-related diseases. According to the National Council on Aging, 94.9% of adults aged 60 and older have at least one chronic condition, and 78.7% have two or more [1]. This trend poses a significant challenge, with high costs from medical treatment and reduced productivity.

Aging affects the entire body, but increasing attention is being given to gut dysbiosis—a condition characterized by an imbalance in gut microbiota, which has been linked to serious diseases, including dementia [2, 3]. Dementia, affecting 57.4 million people in 2019 and projected to reach 152.8 million by 2050 worldwide, is a major burden on older adults [2]. The global economic cost of this condition is estimated at 1.3 trillion USD per year, in addition to the significant unpaid care provided by informal caregivers [4]. Given the potential role of gut dysbiosis on dementia onset, we propose that preventive strategies should focus on maintaining gut microbiota balance. However, recognizing that a single treatment is unlikely to address all the factors contributing to the aging central nervous system’s (CNS) susceptibility to neurodegeneration, we suggest a complementary strategy: enhancing Klotho levels in the choroid plexus (ChP). Although the impact of gut dysbiosis on the CNS has been extensively investigated, its specific effects on the ChP remains comparatively understudied. Equally overlooked are the dynamic fluctuations in Klotho expression at this crucial CNS-periphery interface. Addressing this dual knowledge gap—linking gut microbiota-induced alterations to changes in choroid plexus (ChP) function and Klotho regulation—not only reveals a previously underexplored dimension of neuroimmune crosstalk, but also paves the way for innovative therapeutic strategies. One such approach, proposed in this narrative review, involves the combined administration of probiotics capable of “rejuvenating” the gut microbiota, along with vectors encoding Klotho cDNA specifically targeting the ChP, as a means to preserve the structural and functional integrity of the aging brain.

## The gut-brain axis

The gut microbiota is composed of over 40 trillion microbial organisms, comprising bacteria, viruses, protozoa, fungi, and archaea. Its metagenome is often referred to as the “third mammalian genome,” complementing the nuclear and mitochondrial genomes [5]. It contains over 3 million genes that encode thousands of molecules [6] and plays a crucial role in fundamental processes, including the metabolism of dietary components [7], the production of vitamins, tryptophan, and polyphenol metabolites [8], the regulation of immune defense [9], and protection against pathogens [10]. Not surprisingly, gut dysbiosis can contribute to the onset of various diseases, not only intestinal (e.g., colorectal cancer, inflammatory bowel disease) but also systemic (e.g., type 2 diabetes, chronic kidney disease, autoimmune and rheumatic diseases) [11–15]. Particularly intriguing is the evidence of a bidirectional interaction between the gut and the CNS, mediated by the movement of microorganisms and their byproducts, as well as by the parasympathetic nervous system that innervates the gut [16]. Studies have also identified “neuro-metabolites”—molecules such as serotonin and γ-aminobutyric acid—that are produced by the gut microorganisms or by intestinal epithelial cells in response to gut microbiota stimulation, and can directly influence the CNS [8].

During aging, the gut microbiota experiences a reduction in biodiversity, a decline in beneficial microbes like *Bifidobacterium* and *Lactobacillus*, and an increase in potentially harmful taxa such as *Proteobacteria* and *Bacteroidetes* [17]. This leads to chronic inflammation due to processes such as disruptions in short-chain fatty acid (SCFA) synthesis, aberrant catabolism of dietary components, weakening of the ability to prevent the colonization of harmful species, and alterations in redox balance [18, 19]. An interesting study conducted in healthy primates, which helps eliminate confounding variables such as lifestyle and medication that can bias results in human cohorts, identified another age-related change: a significant decline in network connectivity, indicating a reduction in microbial interactions [20]. Despite ongoing research, the mechanisms by which alterations in the gut microbiota negatively impact the development of severe neurological disorders, including Alzheimer’s and Parkinson’s diseases [21, 22], remain incompletely understood. One hypothesis suggests that gut dysbiosis triggers enhanced intestinal barrier permeability, commonly referred to as “leaky gut” syndrome [23], leading to systemic inflammation through microbial invasion of the mesenteric lymphoid tissue [24]. A potential consequence is the impairment of both the blood–brain barrier (BBB) and the blood-cerebrospinal fluid barrier (bCSFB) [25, 26], compromising the brain’s ability to maintain a controlled environment and allowing infiltration of harmful blood-borne substances, including toxins and pathogens. This may lead to neuroinflammation, neuronal damage, and cognitive decline [27]. Other factors might also play a role. For instance, germ-free mice exhibit reduced levels of serotonin in the brain [28]. Altered serotonin levels can negatively affect not only brain synapse formation and plasticity but also the enteric nervous system, potentially creating a vicious cycle [29]. However, regardless of what is well established and what remains to be proven, it is clear that taking proactive measures to preserve gut microbiota homeostasis could be a significant achievement in prolonging CNS health.

## The relationship between ChP and gut microbiota

The ChP is a complex structure located in the ventricles, consisting of fenestrated capillaries surrounded by stroma and a monolayer of cuboidal ciliated epithelial cells. These cells are directly responsible for CSF secretion and clearance, and form a semi-permeable barrier that allows molecules up to 70 kDa to pass through [30]. The ChP also hosts resident macrophages [31] and is one of the few sites within the CNS where T cells are present [32], contributing to immunosurveillance. Evidence suggests that the epithelial cells of the ChP detect changes in peripheral inflammatory status, which in turn alters their function. This includes increased release of extracellular vesicles and pro-inflammatory miRNAs [33], as well as the production of trafficking molecules that facilitate interactions with CD4^+^ T lymphocytes [34].

Despite its essential role in maintaining brain homeostasis, ChP remains relatively overlooked, particularly in terms of its interactions with the gut microbiota. A recent study highlighted a potential involvement of the gut microbiota in ensuring the optimal permeability of the ChP: in mice depleted of gut microbiota through antibiotic treatment, Xie and colleagues [26] found that 79 genes were differentially expressed in the ChP compared to control animals. Among the downregulated genes, 13 were involved in barrier function, resulting in the suppression of the Gene Ontology categories related to cell junctions, cell adhesion, and membrane components (both integral and intrinsic). The gut microbiota may also contribute to maintaining the structural integrity of the ChP as evidenced by the disruption of zonula occludens-1 tight junction protein network in the ChP epithelium of germ-free mice [35]. Despite Knox and colleagues [35] finding no changes in the vascular network, a fundamental link has been identified between the intestinal vascular barrier (IVB) and the vascular barrier of the ChP (ChP-VB). In response to increased permeability of the IVB due to inflammatory processes, the ChP-VB reduces its own permeability by regulating plasmalemmal vesicle–associated protein-1, an endothelial cell-specific membrane protein involved in the formation of caveolar diaphragms [30]. Notably, increased permeability of ChP capillaries during gut dysbiosis may facilitate the entry of pro-inflammatory mediators into the cerebrospinal fluid, further exacerbating the effects of dysbiosis. Interestingly, in addition to the ChP itself, its macrophages are closely dependent on the gut microbiota as well. Among non-parenchymal CNS-associated macrophages, those in the ChP are the most sensitive, in terms of transcriptional profiles and numbers, to the composition of the gut microbiota, with a significant influence on the altered immune response observed in germ-free mice [36].

An even greater information gap exists regarding the relationship among the ChP, gut microbiota, and age. To the best of our knowledge, only one study has directly analyzed this topic. Cruz-Pereira and colleagues [37] found that treating late-adult mice with antibiotics resulted in a reduction of CD4^+^ T cell accumulation in the ChP and a reversal of social behavior deficits. Much remains to be understood.

## The ChP and Klotho

Klotho is a transmembrane enzyme related to β-glucuronidases that functions as a coreceptor for fibroblast growth factor 23 (FGF23). It can be cleaved by proteases like A Disintegrin and Metalloproteinases 10 and 17 [38], producing a soluble form (s-Klotho) primarily found in body secretions including blood, urine, and CSF. Membrane and s-Klotho together regulate essential metabolic processes, such as mineral and energy metabolism, calcium homeostasis, and synaptic function [39, 40]. Moreover, Klotho acts as a potent antioxidant [41], counteracts vascular calcification, and reduces arterial stiffness [42]. Notably, Klotho is widely regarded as an anti-aging factor because, independently of FGF23, it inhibits several pathways associated with aging and age-related diseases. Specifically, it reduces the activity of (i) transforming growth factor-β (TGF-β), a pleiotropic cytokine known for promoting cellular senescence, stem cell decline, immunological impairment, and fibrosis; (ii) insulin-like growth factor 1 (IGF-1), involved in the insulin/IGF-1 pathway, which plays a role in cancer progression and, through FOXO inhibition, impedes the synthesis of numerous antioxidant enzymes; (iii) the Wnt pathway, which triggers cell senescence, reduces stem cell survival, and collaborates with TGF-β to induce fibrosis when excessively activated; and (iv) NF-κB, a transcription factor that prompts immune and inflammatory responses, inhibits apoptosis of immune cells, promotes their proliferation, and contributes to vascular lesions [43].

Aging leads to a decline in Klotho expression. In human studies, older adults have significantly lower concentration of s-Klotho compared to younger adults, and this reduction is strongly correlated with the onset of age-related conditions, including chronic kidney disease and cardiovascular disease [44, 45]. Murine models of various aging-related pathologies have been extensively studied, showing a consistent decline in s-Klotho levels both systemically and in the brain [46]. Notably, Klotho overexpression in mice has been shown to extend lifespan and significantly improve learning and memory [47]. Fewer data exist for naturally aged wild-type animals [48, 49], highlighting a critical need to investigate the mechanisms linking physiological changes—often observed even with maintained physical and cognitive fitness—to the development of pathological conditions. However, what is certain is that reduced Klotho levels impair autophagy, increase oxidative stress, and promote neuroinflammation; this decline also compromises the integrity of bCSFB making the brain further susceptible to the development of pathological phenomena [46]. Particularly impactful could be the reduction of Klotho produced by the ChP, not only because it is the primary source of Klotho alongside the kidneys, but also because it likely produces s-Klotho for the CSF [50]. Using a murine model with selective knockout of Klotho in the ChP (Klotho^flox/flox^), researchers have shown increased expression of multiple pro-inflammatory factors and triggered macrophage infiltration in this structure, promoting immune-mediated neuropathogenesis [51].

## The role of aquaporins in the ChP and their interaction with Klotho

CSF synthesis largely depends on the water channel Aquaporin-1 (AQP1) [52]. Predominantly expressed in ChP epithelial cells, AQP1 facilitates rapid water transport, which is essential for CSF formation [53–55]. Supporting AQP1’s critical role, AQP1-null mice exhibit a 20–56% reduction in CSF pressure and production [56]. Recently, it has been shown that aging leads to a significant decline in AQP1 expression at both the mRNA and protein levels [48], impacting fluid balance as well as impairing metabolic waste clearance and the distribution of essential neurotrophic factors [57, 58]. This decline may contribute to neurodegenerative processes.

Beyond AQP1, AQP4 has gained interest in the context of brain aging. AQP4 is primarily found in astrocytic endfeet and plays a key role in clearing metabolic waste products through the glymphatic system [59–61]. Specifically, the extended isoforms of AQP4 (AQP4ex), generated through a translational readthrough mechanism [62], are crucial for maintaining the polarization of AQP4 channels at the astrocytic perivascular domain [63], thereby ensuring optimal efficiency of the glymphatic waste clearance system. Notably, AQP4 significantly interacts with Klotho [64–66]. Klotho has been widely shown to suppress P38 mitogen-activated protein kinase (MAPK) activation in various pathological conditions, including neurodegenerative diseases [67–70], thereby reducing the consequences of its activation, such as brain edema, BBB disruption, neuroinflammation, and programmed cell death [71]. Using an animal model of ischemic injury, Zhu and colleagues [64] demonstrated that overexpression of the Klotho gene, specifically through inhibition of the p38 MAPK signaling pathway, influences AQP4 synthesis, suggesting that Klotho may exert a protective role by modulating AQP4 levels and astroglia viability. Increasing Klotho levels could provide an additional preventive and therapeutic effect by enhancing glymphatic function and reducing AQP4 mislocalization—two conditions associated with aging and neurodegenerative diseases—possibly through the modulation of AQP4ex function.

**Fig. 1:**
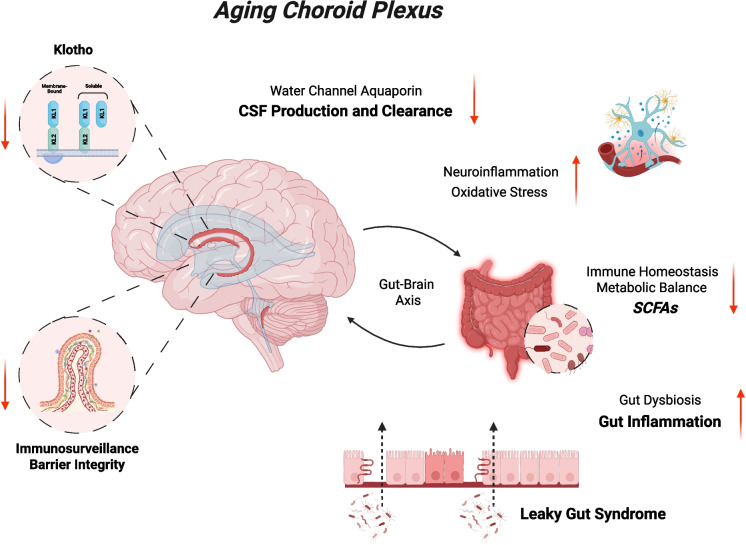
Schematic representation of the effects of gut dysbiosis and reduced Klotho levels on the aged choroid plexus (ChP). During aging, the gut microbiota undergoes changes in composition and function, a condition known as gut dysbiosis. This results in altered nutrient processing and reduced production of essential molecules (e.g., lower levels of short-chain fatty acids, SCFAs), accompanied by a decline in immune protection, increased local inflammation, and a significant weakening of the gut’s selective barrier function (a condition known as “leaky gut” syndrome), ultimately leading to systemic inflammation and neuroinflammation. Aging is also associated with a reduction in Klotho levels, particularly in the ChP, one of the main sources of this protein. The combination of gut dysbiosis and reduced Klotho synthesis causes significant impairment of the ChP’s structural integrity and immune function—partly due to microglial dysregulation—accompanied by decreased cerebrospinal fluid (CSF) production and clearance, as well as increased inflammation and oxidative stress. The image was created using the BioRender software

Summarizing, despite the limited data available so far, the ChP emerges as a key hub for age-related changes that potentially impact the entire brain (Fig. 1), making it an important target for potential intervention strategies, including Klotho upregulation, as illustrated in the following paragraphs.

## Probiotics as preferential modulators of the gut microbiota

Several approaches have been studied to modulate the gut microbiota and to counteract age-related cognitive decline by leveraging the gut-brain axis, including nutritional interventions [72, 73], prebiotics and probiotics [74, 75], and fecal microbiota transplantation (FMT) [76, 77]. Among the possible intervention protocols, probiotic use appears to be one of the most promising. Compared to FMT, which is used only for *Clostridioides difficile* infection due to problems in maintaining eubiosis afterward [78], probiotics are easier to implement. Nutritional interventions are challenging to monitor long-term, and prebiotics offer shorter-lasting effects compared to probiotics (which are living microorganisms), as their impact on the gut microbiota is hard to predict [79]. Probiotics enhance microbial community stability, produce postbiotics like SCFAs, and modulate immune responses [80, 81]. Derived from the fermentation of indigestible dietary fibers, SCFAs—primarily acetate, propionate, and butyrate, which together account for approximately 95% of the total SCFAs in the human colon [82]—serve as critical energy substrates, regulate inflammatory pathways via G-protein coupled receptors, and preserve both epithelial and endothelial barrier integrity [83–85]. Notably, butyrate enhances the expression of the tight junction protein Occludin, thereby improving BBB function [86].

Aging is associated with a significant decline in SCFA-producing bacteria, particularly butyrate- and propionate-producing strains, along with an increase in opportunistic and potentially proinflammatory commensal microbes [17, 87, 88]. Research has consistently shown that an imbalance in SCFA production is associated not only with physiological aging [89] but also with neurological disorders, including Alzheimer’s and Parkinson’s diseases [27, 90–92]. Among the primary SCFA-producing genera are *Lactobacillus*, *Bifidobacterium*, and *Clostridium*. Strains from *Lactobacillus* and *Bifidobacterium*, in particular, are key contributors to gut-brain axis homeostasis through their ability to lower systemic inflammation and reinforce tight junctions. For instance, *Lactobacillus rhamnosus* modulates cytokines such as TNF-α and IL-6 [93], indirectly bolstering BBB function [94]. Similarly, *Bifidobacterium longum* and *Bifidobacterium breve* help preserve barrier integrity by restoring SCFAs levels as well as diminishing oxidative stress and glial activation [95–97]. Next-generation probiotics are increasingly studied for their unique metabolic and immunomodulatory properties [98]. Key species include *Akkermansia muciniphila*, *Faecalibacterium prausnitzii*, and *Roseburia intestinalis* [99]. *Akkermansia muciniphila* supports epithelial and endothelial integrity, potentially enhancing CNS barrier function [100]. In an Alzheimer’s disease mouse model, it reduced amyloid-beta (Aβ) plaque deposition, improved glucose homeostasis, and alleviated cognitive deficits, highlighting its neuroprotective potential [101]. *Faecalibacterium prausnitzii* exhibits strong anti-inflammatory effects by modulating immune responses and inhibiting NF-κB activation through secreted metabolites [102]. It also shows neuroprotective properties in a treatment-resistant depression model, lowering proinflammatory cytokines (IFN-γ, TNF-α, CRP, IL-6), restoring neurotransmitter balance, and increasing Brain-Derived Neurotrophic Factor expression [103]. *Roseburia intestinalis* is a key butyrate producer. Its supplementation has been associated with reduced neuroinflammation, serotonergic modulation, and alleviation of depression-like behaviors [104]. Its decrease in neurodegenerative diseases, including Parkinson’s, further supports its neuroprotective potential [104, 105].

Outlining, since both the BBB and ChP rely on the integrity of tight junctions and are susceptible to inflammatory and metabolic disturbances, it is reasonable to hypothesize that conventional and next-generation probiotics beneficial to the BBB and systemic chronic inflammation could also enhance ChP barrier function.

## Klotho upregulation as a therapeutic strategy for neurodegeneration

Evidence suggests that exogenous upregulation of Klotho in the CNS could positively affect cognition. For instance, a study by Zeng and colleagues [106] demonstrated that Klotho upregulation in 10-month-old APP/PS1 mice, a model of genetically induced dementia, reduced Aβ burden and partially restored cognitive performance. Similarly, another study using the same murine model showed that delivery of full-length Klotho cDNA reduced Aβ accumulation, enhanced synaptic plasticity, and improved cognitive function [65].

It is important to emphasize that endogenous Aβ proteins have vital physiological roles, such as promoting synaptic plasticity in hippocampal neurons [107] and exhibiting antimicrobial activity [108]. Their indiscriminate reduction could lead to side effects. However, Klotho upregulation appears to stimulate Aβ clearance, thereby preventing excessive Aβ accumulation and preserving its homeostasis [65, 106], aligning with recent studies suggesting this as the safest and most effective approach [109].

Notably, the positive effects associated with brain upregulation of Klotho seem significantly mediated by the enhancement of ChP function. The increase in Klotho levels in ChP preserves CSF production and improves the exchange of molecules between blood and CSF by strengthening tight junctions and reducing inflammatory signaling [51]. Therefore, to counteract brain aging, a promising approach is increasing Klotho levels through gene-based delivery strategies that selectively target the ChP [110]. Since intracranial administration is not feasible in clinical settings, intravenous or intranasal delivery must be adopted, making selectivity the most critical challenge.

Choosing the right vector is crucial for a successful first attempt. According to a recent comparative study, both lentiviruses and adeno-associated viruses (AAV) can selectively infect ChP epithelial cells. Nevertheless, the former exhibits relatively low transduction efficiency and an immunogenic profile, whereas the latter—particularly AAV2/5 and AAV2/8—demonstrates superior transduction efficiency, making AAV ideal for targeted gene delivery in ChP [110]. Adenoviral vectors, though explored for ChP gene delivery, are less suitable due to their broader tropism, affecting ependymal cells and other CNS regions [111]. An additional step forward can be made by using promoters with high activity in ChP epithelial cells, such as the Transthyretin (*TTR*) promoter. TTR is a homotetrameric thyroid hormone transport protein, primarily synthesized by hepatocytes and the ChP epithelium, and secreted into both plasma and CSF [112, 113]. When carrying a specific 3 kb upstream region, *TTR* is expressed in the ChP but not in other brain areas. However, there are some issues. The 3 kb *TTR* promoter is less potent than the full set of endogenous *TTR* regulatory elements, likely because it omits key enhancer regions [114]. Furthermore, systemic delivery can lead to potential off-target expression in the liver. One way to mitigate this issue is through miRNA de-targeting. By incorporating liver-enriched microRNA recognition sequences (e.g., miR-122a and miR-199a) into the gene construct at the UTR region, the therapeutic transcript can be degraded in hepatic cells while remaining stable in the ChP, where these miRNAs are absent [115]. Another strategy involves the Multiplex-CREATE (M-CREATE) method that has led to the development of engineered AAV capsids optimized for intravenous delivery, enhancing CNS targeting while minimizing off-target expression [116]. Nevertheless, the limited packaging capacity of AAV (about 4.7 kb, including its inverted terminal repeats) can pose a significant challenge when using large constructs, such as full-length Klotho in combination with the 3 kb *TTR* promoter. Solutions include employing smaller *TTR* promoter fragments and truncated Klotho isoforms, albeit with suboptimal therapeutic potential. Dual AAV vector strategies have emerged as a promising approach to bypass packaging constraints, enabling the delivery of larger transgenes. A large therapeutic gene is divided into two separate AAV cassettes, one containing the promoter and 5′ portion of the gene, and the other carrying the remaining coding sequence and polyadenylation signal. Upon co-transduction into the same cell, these cassettes recombine—often via splicing of an engineered junction—to reconstitute the full-length transcript [117]. A notable advancement in this field is the REVeRT dual AAV strategy, which utilizes mRNA trans-splicing to efficiently reconstitute large genes in vivo, providing greater flexibility in split site selection and enhancing transgene expression in multiple organs, including the brain [118]. However, significant challenges remain in terms of recombination efficiency, sustained expression, and potential unintended “alien” protein production, which may impact therapeutic outcomes. Further optimization is mandatory before dual-vector systems can be reliably applied to ChP-targeted gene therapy.

Overall, although significant technical limitations still exist, the foundations have been laid, and the prospects for achieving upregulation in the ChP appear promising.

## Conclusions

**Fig. 2:**
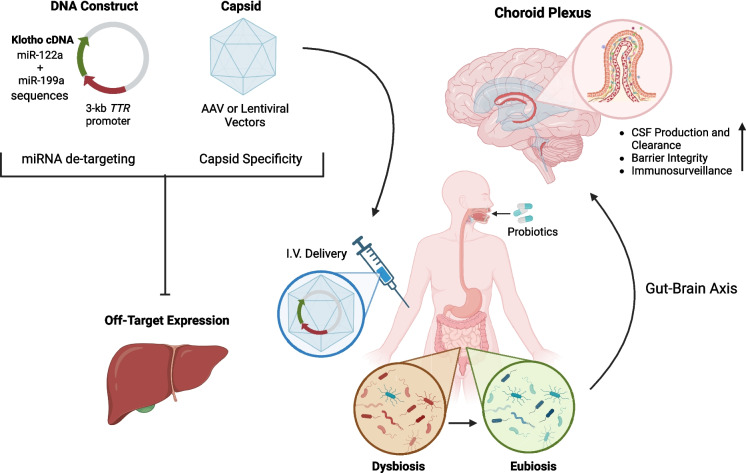
Restoring gut microbiota eubiosis and recovering Klotho levels in the choroid plexus (ChP) as a potential preventive strategy for the onset of neurodegenerative phenomena in older adults. The administration of probiotics, particularly those genera that synthesize short-chain fatty acids, such as *Lactobacillus* and *Bifidobacterium*, not only helps counteract gut microbiota dysbiosis but also influences the gut-brain axis. These probiotics can reduce inflammation and strengthen tight junctions, thereby improving the structure and function of barriers, including the ChP. On the other hand, upregulating Klotho levels in the ChP would have a cumulative effect on maintaining barrier integrity, cerebrospinal fluid (CSF) production and clearance, and immunosurveillance. Ensuring selective Klotho synthesis in the ChP is crucial. At the construct level, the Transthyretin (*TTR*) promoter can be utilized, incorporating liver-enriched microRNA recognition sequences to target degradation in hepatic cells (e.g., miR-122a and miR-199a). Regarding the viral capsid, adeno-associated viruses (AAVs) can selectively target the ChP, particularly when engineered using the Multiplex-CREATE (M-CREATE) method. The image was created using the BioRender software

According to several researchers, the ChP is a pivotal point of communication that links the periphery to the brain, acting as the interface between blood and CSF [119]. In recent years, the ChP has emerged as one of the most sensitive targets for both gut dysbiosis and the reduction of Klotho levels during aging. These factors have consequences not only for the function of the ChP (i.e., reduced barrier capacity, interference with CSF production and clearance, altered immunosurveillance) [45, 120], but also for the brain as a whole (e.g., neuroinflammation, oxidative damage, reduced vascular support). Surprisingly, little has been done to make the ChP a therapeutic target. In this context, we propose a cutting-edge protocol that combines two approaches aimed at triggering an endogenous response to restore cerebral homeostasis through action on the ChP itself (Fig. 2). To our knowledge, this is the first proposal to target the ChP in order to counteract neurodegenerative phenomena that predispose individuals to dementia onset.

However, there are limitations to consider. Technical expertise is still lacking in ensuring precise targeting of systemic gene therapy aimed at restoring Klotho levels in the ChP. A recent study [121] showed that excessive overexpression of processed Klotho, but not secreted Klotho, can disrupt mineral metabolism and bone microstructure, underlining the need for precise control of isoform specificity and expression levels in Klotho-based therapies. In oncology, Klotho may exert either pro- or anti-proliferative effects depending on tissue type, reinforcing the importance of careful therapeutic design [122]. As a result, despite the suggested precautions, off-target expression may still occur, potentially leading to unintended and unpredictable effects—which could be particularly significant in older adults, whose homeostatic capacity is already reduced. Additionally, interactions with the resident microbiota following probiotic administration are not yet fully predictable, which could interfere with their integration and impact the outcome. Therefore, rigorous and well-designed studies in animal models will be critical to optimize delivery strategies, thoroughly assess long-term consequences, and robustly establish the safety and therapeutic potential of the proposed approach as a necessary step toward clinical translation.
